# Diabetic kidney disease within the framework of cardio-kidney-metabolic health: a paradigm shift

**DOI:** 10.3389/fphar.2026.1907928

**Published:** 2026-09-08

**Authors:** Juarez R. Braga, Fatima Ayub, Srikanth Vallurupalli, Nithin Karakala, Joseph H. Holthoff

**Affiliations:** 1 Division of Nephrology, Central Arkansas Veterans Health System, Little Rock, AR, United States; 2 Department of Nephrology, University of Arkansas for Medical Sciences, Little Rock, AR, United States; 3 Department of Cardiology, University of Arkansas for Medical Sciences, Little Rock, AR, United States

**Keywords:** cardio-kidney metabolic syndrome, cardiovascular outcome, chronic kidney disease, diabetic kideny disease, renoprotective therapy

## Abstract

Diabetic kidney disease (DKD) is the most common cause of chronic kidney disease (CKD) and kidney failure in addition to determining an elevated risk of cardiovascular (CV) disease. Traditional approaches to DKD management have focused primarily on slowing kidney disease progression through improved glycemic control. However, growing recognition of the complex interplay among metabolic, kidney, and CV dysfunction has led to the emergence of the cardio-kidney-metabolic (CKM) syndrome framework, emphasizing the interconnected pathophysiology and shared risk profiles of these conditions. This evolving paradigm has transformed the understanding and management of DKD from an isolated kidney disorder to a multisystem disease requiring integrated therapeutic strategies. Recent advances have significantly altered the landscape of DKD management. In addition to angiotensin-converting enzyme inhibitors and angiotensin receptor blockers, newer therapies including sodium-glucose cotransporter 2 inhibitors, glucagon-like peptide-1 receptor agonists, and non-steroidal mineralocorticoid receptor antagonists have demonstrated substantial kidney and CV protective effects. Emerging evidence suggests that combination therapy may potentially shift the treatment paradigm from slowing CKD progression toward preservation of kidney function. In this review, we discuss DKD within the broader framework of CKM syndrome, highlighting the epidemiologic, pathophysiologic, prognostic, and therapeutic intersections among diabetes, CKD, and CV disease. We summarize major therapeutic advances, review evidence supporting multidrug combination strategies, and examine current gaps between clinical evidence and real-world implementation. We further propose a phenotype-driven framework for sequencing combination therapy based on each patient’s dominant pathophysiologic driver as a hypothesis-generating alternative to uniform add-on strategies. Finally, we discuss the future direction of DKD management and the need for integrated multidisciplinary approaches aimed at improving outcomes.

## 1 Introduction

Diabetes and chronic kidney disease (CKD) are major public health problems worldwide, and the number of people affected by both conditions continues to climb. Projections estimate that more than 1.3 billion individuals will have diabetes by 2050, largely driven by increasing rates of obesity, and more than 40% of countries are expected to have a diabetes prevalence exceeding 10% (Ong et al., 2023). Similarly, CKD currently affects nearly 800 million individuals worldwide, representing more than 14% of the adult population (Mark et al., 2025). Diabetic kidney disease (DKD) represents the clinical and pathophysiologic intersection between diabetes, CKD, and cardiovascular (CV) disease. DKD remains the most common etiology of CKD and accounting for approximately 40% of end-stake kidney failure (ESKD) cases (United States Renal Data System, 2025). However, the burden of DKD extends well beyond progression to kidney failure. Individuals with DKD are also at high-risk of CV morbidity and mortality. In fact, people with CKD more often die of CV disease than survive to reach ESKD. This excess risk is particularly pronounced among younger individuals (Nyeland et al., 2023).

Furthermore, individuals with diabetes have been described as having an overall mortality risk twice that of the general population, a risk comparable to that of individuals without diabetes who have experienced a prior myocardial infarction (Choi et al., 2026). Importantly, emerging evidence suggests that this heightened CV risk associated with diabetes is largely driven by the subgroup of patients with concomitant kidney disease, underscoring the close interplay among metabolic dysfunction, CKD, and CV disease (Afkarian et al., 2013). As these interconnections have come into focus, both the conceptualization and treatment of DKD have shifted fundamentally. No longer regarded as a potential complication of diabetes, DKD is increasingly understood as a systemic disorder. Accordingly, the therapeutic objective in DKD is shifting from slowing CKD progression toward ‘integrated cardiorenal-metabolic protection’ with preservation of kidney function and simultaneous reduction of CV risk. Throughout this review, we use ‘integrated cardiorenal-metabolic protection’ to refer to a therapeutic strategy that simultaneously targets shared pathophysiologic pathways across the kidney, CV, and metabolic systems as opposed to single-organ-focused treatment with the goal of reducing composite risk across all three domains rather than optimizing any single outcome in isolation.

In this review, we critically appraise the evidence behind concepts such as cardio-kidney-metabolic health and CKD remission and the practical implementation barriers that determine whether therapeutic advances reach patients. In addition, we propose a phenotype-driven framework for sequencing combination therapy for DKD, in which the dominant pathophysiologic driver in each patient informs which agent is prioritized first, rather than defaulting to a uniform add-on sequence.

## 2 The construct of cardio-kidney-metabolic syndrome

Recognition of the interactions among diabetes, CKD, obesity, and CV disease has led the American Heart Association to propose the concept of cardio-kidney-metabolic (CKM) syndrome (Ndumele et al., 2023). CKM syndrome is defined as a systemic disorder driven by interconnected metabolic, kidney, and CV pathways that collectively contribute to progressive morbidity and mortality. Within this framework, individuals with DKD would generally be classified as having stage 2 CKM syndrome or higher, reflecting the coexistence of metabolic dysfunction and established kidney disease (Table 1). Although many have criticized the CKM framework, the epidemiologic and mechanistic overlap among diabetes, CKD, and CV disease justifies the need for a unifying framework that better reflects the interdependence of these conditions. In line with this concept, the American Heart Association recently modified its CV disease risk calculator and released the Predicting Risk of Cardiovascular Disease Events (PREVENT) to accommodate kidney factors (Khan et al., 2024). The main difference between the PREVENT calculator and the ACC Pooled Cohort Equations was the incorporation of estimated glomerular filtration rate (eGFR) and urinary albumin-to-creatinine ratio (UACR) as independent CV risk factors, recognizing CKD as an independent determinant of risk. Both eGFR and UACR have demonstrated dose-dependent associations with kidney and CV outcomes (Matsushita et al., 2010; Claudel and Verma, 2025). The risk of all-cause mortality and CV mortality rises substantially as eGFR declines below 75 mL/min/1.73 m^2^ (Writing Group for the CKDPC et al., 2023). Similarly, albuminuria has emerged as a continuous marker of risk as opposed to a categorical variable. Even low-grade elevations below the traditional threshold of 30 mg/g are associated with increased risks of CKD progression, CV disease, and mortality (Verma et al., 2024; Tanaka et al., 2016). Importantly, reductions in albuminuria are associated with improved kidney and CV outcomes (Heerspink et al., 2019; Levey et al., 2020). Although the incremental improvement in CV risk prediction achieved by incorporating eGFR and UACR into multivariable risk equations is modest, the significance lies in reframing DKD within the CKM construct.

**TABLE 1:** Stages of cardio-kidney-metabolic syndrome as proposed by the American heart association.

Stage	Definition	Conditions
0	No risk factors	Normal abdominal circumference, BMI, glucose, blood pressure, lipids
		No evidence of CKD
		No evidence of subclinical or clinical CVD
1	Excess/dysfunctional adipose tissue	Overweight/obesity
		Abdominal obesity
		Impaired glucose tolerance or prediabetes
2	Metabolic risk factors and CKD	Hypertension
		Diabetes
		Hypertriglyceridemia
		Metabolic syndrome
		Moderate- to high-risk CKD
3	Subclinical CVD	Subclinical ASCVD
		Subclinical HF
		Very high-risk CKD
		High predicted risk for CVD
4a	Clinical CVD	CAD
		HF
		Stroke
		AFib
		PAD
4b	Clinical CVD + kidney failure	Any type of CVD
		Kidney failure

AFib: atrial fibrillation; ASCVD: atherosclerotic cardiovascular disease; BMI: body mass index; CAD: coronary artery disease; CKD: chronic kidney disease; CVD: cardiovascular disease; HF: heart failure; PAD: peripheral arterial disease.

## 3 Major therapeutic advances for diabetic kidney disease

**FIGURE 1:**
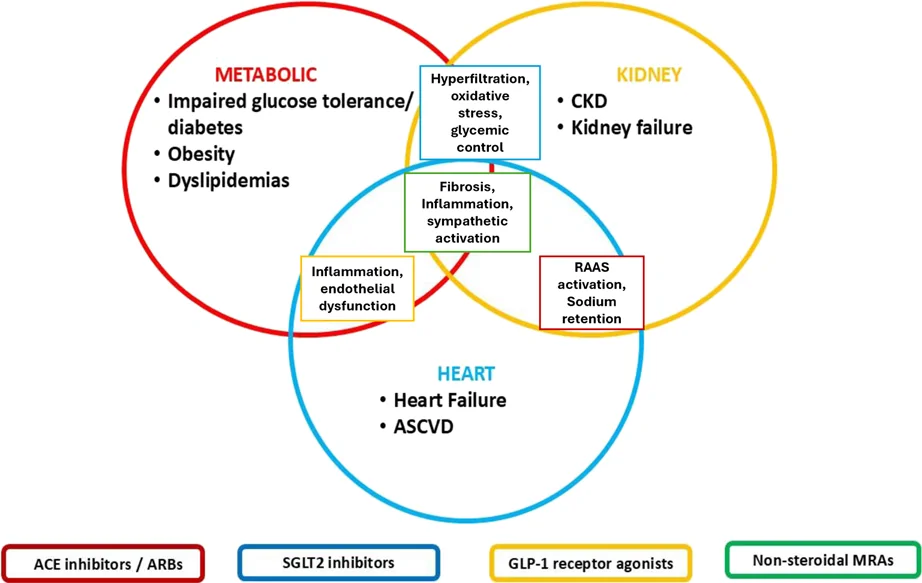
Interconnection between cardiovascular, kidney, and metabolic disease and drugs classes that offer cardio-kidney protection. ACE, angiotensin-converting enzyme; ARB, angiotensin-receptor blocker; ASCVD, atherosclerotic cardiovascular disease; CKD, chronic kidney disease; ESKD, end-stage kidney disease; GLP-1, glucagon-like peptide; MRA, mineralocorticoid receptor antagonist.

The broader CKM construct is supported not only by the epidemiology of diabetes and CKD but also by the major therapeutic advances over the past decade that simultaneously improve kidney and CV outcomes. Historically, management of DKD focused primarily on glycemic control. However, emerging evidence has demonstrated that DKD progression is driven by multiple interconnected mechanisms including glomerular hypertension, endothelial dysfunction, inflammation, oxidative stress, metabolic dysregulation, and fibrosis. This evolving understanding has led to the development of therapies targeting complementary pathophysiologic pathways, including sodium-glucose cotransporter 2 (SGLT2) inhibitors, glucagon-like peptide-1 receptor agonists (GLP1-RAs), and mineralocorticoid receptor antagonists (MRAs), in addition to angiotensin-converting enzyme (ACE) inhibitors and angiotensin receptor blockers (ARBs) (KDIGO, 2022). Rather than acting on isolated organ systems, these therapies exert overlapping cardiorenal protective effects across the broader CKM axis, supporting a paradigm shift toward integrated multidrug cardiorenal-metabolic protection (Figure 1).

### 3.1 Angiotensin-converting enzyme inhibitors/angiotensin receptor blockers

ACE inhibitors and ARBs primarily exert kidney protection through modulation of intraglomerular hemodynamics by preferential efferent arteriolar vasodilation, thereby reducing glomerular capillary hypertension and proteinuria. In addition to their hemodynamic effects, renin-angiotensin system blockade also attenuates inflammatory and profibrotic signaling pathways involved in DKD progression. Across the 1990s and 2000s, ACE inhibitors and ARBs became the first drug classes which demonstrated meaningful kidney protection in DKD, and they are now the background therapy onto which today’s cardiorenal therapies are added to. Published in 1993, the first ACE inhibitor trial to demonstrate benefit in DKD compared captopril with placebo in approximately 400 patients who had insulin-dependent diabetes and urinary protein excretion of at least 500 mg per day. Relative to placebo, captopril lowered the risk of the primary endpoint, doubling of baseline serum creatinine, by 48% (95% confidence interval [CI], 16%–69%) (Lewis et al., 1993). The first trial with an ARB was published in 2001, RENAAL trial, and it examined the effects of losartan among 1,500 individuals with diabetic nephropathy. The trial showed a 16% risk reduction in the incidence of doubling of the serum creatinine, end-stage kidney failure, as well as death as compared to placebo (Brenner et al., 2001). These studies established renin-angiotensin system blockade as the standard background therapy for DKD. Since then, multiple studies and meta-analyses have confirmed the reno-protective effects of ACE inhibitors and ARBs in DKD. A recent systematic review and metanalysis summarizing one hundred and nine studies and 28,341 randomized participants with DKD revealed that relative to placebo or no treatment, ACE inhibitors reduced the incidence of kidney failure (8 studies, 6643 participants: relative risk [RR] 0.61, 95% confidence interval [CI] 0.39 to 0.94; I^2^ = 0%; low certainty). ARBs likewise lowered the risk of kidney failure (3 studies, 3227 participants: RR 0.82, 95% CI 0.72 to 0.94; I^2^ = 0%; low certainty), of serum creatinine doubling (4 studies, 3280 participants: RR 0.84, 95% CI 0.72 to 0.97; I^2^ = 32%; low certainty), and of progression from microalbuminuria to macroalbuminuria (5 studies, 815 participants: RR 0.44, 95% CI 0.23 to 0.85; I^2^ = 74%; low certainty). However, unlike more contemporary therapies, the CV benefits of ACE inhibitors and ARBs appear more modest and less consistent across studies. Compared to placebo or no treatment, ACE inhibitors may not offer a benefit in terms of all‐cause death (24 studies, 7413 participants: RR 0.91, 95% CI 0.73 to 1.15; I^2^ = 23%; low certainty) as well as ARBs in relation to all‐cause death (11 studies, 4260 participants: RR 0.99, 95% CI 0.85 to 1.16; I^2^ = 0%; low certainty) and CV death (6 studies, 878 participants: RR 3.36, 95% CI 0.93 to 12.07; low certainty) (Natale et al., 2024).

### 3.2 SGLT2 inhibitors

Beyond their glucose-lowering action, SGLT2 inhibitors confer substantial cardiorenal protection by restoring tubuloglomerular feedback, lowering intraglomerular pressure, promoting natriuresis, enhancing metabolic efficiency, and dampening inflammatory and oxidative-stress pathways. Critically, these benefits are not contingent on glycemic improvement and hold across a wide spectrum of kidney function and albuminuria. This reduction in intraglomerular pressure via restored tubuloglomerular feedback typically produces an early, dose-dependent decline in eGFR that stabilizes within weeks, a hemodynamic recalibration reflecting successful pressure reduction rather than nephron loss (Ayub and Juncos, 2026). The beneficial effects of this drug class were quantified in a large individual-participant-data meta-analysis, which found that SGLT2 inhibitors lowered the risk of every kidney outcome assessed. Based on 10 randomized trials and over 70,000 participants, the analysis showed a reduction in CKD progression (25.4 vs 40.3 events per 1000 patient-years; hazard ratio [HR], 0.62; 95% CI, 0.57–0.68) that was independent of baseline eGFR and albuminuria. The degree of protection differed by subgroup, but the annual rate of eGFR decline fell across all eGFR and UACR strata, including in the diabetes-only analysis. Kidney failure as a standalone endpoint was likewise reduced (HR, 0.66; 95% CI, 0.58–0.75) (Neuen et al., 2026).

In a separate meta-analysis of 11 trials and 78,607 patients, 42,568 of whom had diabetes at high atherosclerotic CV risk and 15,314 of whom had CKD, SGLT2 inhibitors cut the rate of major adverse CV events by 9% (HR, 0.91; 95% CI, 0.87–0.96; P < 0.0001), with effects that held uniformly across patient populations and every key subgroup. Reduced CV death drove this result (HR, 0.86; 95% CI, 0.81–0.92; P < 0.0001), whereas myocardial infarction was not significantly affected overall (HR, 0.95; 95% CI, 0.87–1.04; P = 0.29) and stroke was unchanged (HR, 0.99; 95% CI, 0.91–1.07; P = 0.77). The CV-death benefit traced mainly to fewer heart failure (HF) deaths and sudden cardiac deaths (HR, 0.68; 95% CI, 0.46–1.02 and HR, 0.86; 95% CI, 0.78–0.95, respectively) and was broadly consistent across subgroups (Patel et al., 2024).

A notable feature of SGLT2 inhibitors is that they confer cardiac protection despite the absence of meaningful SGLT2 expression in the myocardium, indicating that their CV benefits are largely indirect and multifactorial rather than the result of direct myocardial target engagement (Salvatore et al., 2022). By blocking glucose and sodium reabsorption in the proximal tubule, SGLT2 inhibition produces glucosuria and a glycosuria-dependent osmotic diuresis, accompanied by a modest reduction in proximal tubular sodium reabsorption and natriuresis (Yerra and Connelly, 2024). These effects reduce plasma volume and preload, lower arterial blood pressure without a compensatory rise in heart rate, and improve ventricular loading conditions, providing an attractive hemodynamic explanation for the early reduction in HF hospitalizations observed in clinical trials. The explanation, however, has been increasingly questioned and remains an area of active debate. Mechanistic studies suggest that the expected osmotic diuresis is largely offset over time by compensatory, vasopressin-driven renal water conservation, such that the sustained reduction in extracellular volume is smaller than anticipated (Marton et al., 2024). Moreover, the magnitude of clinical benefit does not track with the degree of glycosuria: HF outcomes improve to a similar extent in patients with and without diabetes, despite markedly greater glucose excretion in those with diabetes, and changes in cardiac filling pressures and natriuretic peptides during the initial period of maximal diuresis are small (Packer, 2022). These observations have prompted alternative mechanistic frameworks, most prominently the hypothesis that SGLT2 inhibitors induce a state of nutrient-deprivation signaling that enhances autophagy, restores mitochondrial health, attenuates oxidative and endoplasmic reticulum stress, and reduces proinflammatory and profibrotic signaling within the heart and kidney (Packer, 2022). Additional proposed mechanisms include a shift toward ketone and free fatty acid utilization as a more efficient myocardial fuel, decreasing epicardial fat mass, reductions in cardiomyocyte intracellular sodium and calcium overload with favorable effects on contractility and arrhythmogenicity, and increased erythropoiesis (Lopaschuk and Verma, 2020). The relative contribution of each of these pathways remains incompletely defined, but the collective evidence indicates that the cardiorenal benefits of SGLT2 inhibitors extend well beyond a simple diuretic effect.

### 3.3 GLP1-receptor agonists

GLP1-RAs primarily improve metabolic dysfunction through weight reduction, improved glycemic control but growing evidence also suggests direct anti-inflammatory, anti-atherosclerotic, and renoprotective properties. In a meta-analysis of 12 trials enrolling 17,996 participants with a baseline eGFR below 60 mL/min/1.73m2, GLP-1RAs were significantly associated to a lower risk of the composite kidney outcome, with a weighted event rate of 15.1% versus 17.5% in the placebo arm (odds ratio [OR], 0.85; 95% CI, 0.77–0.94; P = 0.001) and minimal heterogeneity (I^2^<0.01%). The class was similarly associated with reduced risk of eGFR decline exceeding 30% (OR, 0.78; P = 0.004), 40% (OR, 0.76; P = 0.01), and 50% (OR, 0.72; P < 0.001). Across the seven studies reporting all-cause death, among 11,949 patients with baseline CKD, mortality was also lower with GLP-1RAs (OR, 0.77; 95% CI, 0.60–0.98; P = 0.03), albeit with high heterogeneity (I^2^ = 71.6%). Eleven of the 12 studies reported composite CV outcomes specifically in patients with CKD at enrollment; among these 16,368 participants, 2,121 experienced such an event, and the risk was again lower with GLP-1RA use (OR, 0.86; 95% CI, 0.74–0.99; P = 0.03; I^2^ = 40.3%) (Chen et al., 2025).

### 3.4 Mineralocorticoid receptor antagonists

Aldosterone dysregulation and mineralocorticoid receptor (MR) overactivation plays a central role in the progression of DKD. Importantly, pathologic MR activation may persist even in the absence of markedly elevated circulating aldosterone levels due to local intrarenal renin-angiotensin-aldosterone system activation, Rac1 signaling, and non-hemodynamic inflammatory pathways. This evolving understanding has shifted the focus from viewing aldosterone solely as a regulator of sodium and potassium balance toward recognizing MR signaling as an important mediator of cardiorenal injury. Consequently, non-steroidal (nsMRAs), particularly finerenone, have emerged as important therapeutic agents targeting inflammation and fibrotic risk in DKD (Amornritvanich et al., 2025; Jaisser and Farman, 2016; Barrera-Chimal and Jaisser, 2020; Bakris et al., 2020; Pitt et al., 2021). Relative to steroidal MRAs like spironolactone and eplerenone, nsMRAs offer greater receptor selectivity and may carry lower rates of hyperkalemia and off-target adverse effects, all while retaining potent anti-inflammatory and antifibrotic activity. In people with DKD, finerenone reduces albuminuria, slows CKD progression, and improves CV outcomes through pathways that go beyond blood-pressure lowering. Evidence from both experimental and clinical studies indicates that MR blockade enhances endothelial function, dampens macrophage and inflammatory-cell activation, suppresses profibrotic signaling, and limits maladaptive remodeling in the kidney and the CV system (Jaisser and Farman, 2016; Barrera-Chimal and Jaisser, 2020; Bakris et al., 2020). Among patients with CKD and type 2 diabetes already on background RAAS blockade, finerenone in the FIDELIO-DKD trial significantly lowered the risk of kidney failure, sustained eGFR decline, or renal death (Pitt et al., 2021). The FIGARO-DKD trial extended these findings to earlier-stage DKD, where finerenone produced marked reductions in CV events, most notably hospitalization for HF (Agarwal et al., 2022). Reinforcing these trial results, an umbrella review of systematic reviews and meta-analyses found consistent kidney and CV benefits of nsMRAs in people with DKD. Of 8 meta-analyses evaluating nsMRAs in this population, seven found a significantly lower risk of composite kidney events versus placebo, with pooled RRs ranging from 0.76 (0.66–0.88) to 0.86 (0.78–0.95). Five meta-analyses showed that nsMRAs lowered the UACR relative to placebo, and five reported a significantly reduced risk of eGFR decline of at least 40% from baseline (pooled RRs of 0.70 [0.60–0.82] to 0.85 [0.78–0.93]). Two further demonstrated a lower likelihood of initiating renal replacement therapy with nsMRAs than with placebo (pooled RR, 0.80 [0.65–0.99]). Cardiovascular endpoints improved as well: nsMRAs significantly reduced all-cause mortality (pooled RR, 0.90) and composite CV events in DKD (pooled RRs of 0.86 [0.78–0.95] to 0.88 [0.81–0.96]) (Amornritvanich et al., 2025).

## 4 Combination therapy and the evolution toward integrated cardio-kidney-metabolic protection

All those medications have proven benefits in the management of individuals with DKD based on robust randomized controlled clinical trials. Those interventions represent a paradigm shift toward comprehensive CKM protection. However, the addition of multiple therapies to a treatment regimen inevitably increases treatment complexity, cost, pill burden, and the potential for adverse effects. Furthermore, despite the proven efficacy of each individual drug class, the magnitude of benefit achieved through combination therapy and the extent of additive or synergistic effects initially remained uncertain. Nevertheless, the different mechanisms of action of those classes of drugs have led to considerable interest in the potential benefits of combination therapy to decrease the CKM risk. Mechanistically, these therapies exert overlapping but complementary effects. ACE inhibitors and ARBs primarily reduce intraglomerular hypertension and proteinuria through preferential efferent arteriolar vasodilation and attenuation of maladaptive renal renin-angiotensin signaling. SGLT2 inhibitors restore tubuloglomerular feedback, lessen glomerular hyperfiltration, enhance metabolic efficiency, and blunt inflammatory and oxidative-stress pathways. GLP-1RAs, in turn, counter metabolic dysfunction and obesity while also delivering anti-inflammatory, anti-atherosclerotic, and cardioprotective effects. Meanwhile, MRAs target residual inflammatory and profibrotic signaling pathways.

The CONFIDENCE trial provides important evidence that combination therapy is effective in DKD. In that trial, eligible participants had type 2 diabetes, an eGFR of 30–90 mL/min/1.73 m^2^, and albuminuria, defined as a UACR of 100–500 mg/g. They were required to have been on an ACE inhibitor or ARB for more than 1 month at screening and to have avoided any SGLT2 inhibitor in the preceding 8 weeks. Randomization was 1:1:1 to one of three arms: finerenone at 10 or 20 mg daily (with empagliflozin-matching placebo), empagliflozin at 10 mg daily (with finerenone-matching placebo), or both agents combined. The primary outcome was the relative change in log-transformed mean UACR from baseline to day 180. The full analysis population comprised 800 participants, 269 in the combination-therapy arm, 264 in the finerenone arm, and 267 in the empagliflozin arm.

At baseline, more than 97% of participants were receiving ACE inhibitors or ARBs and roughly 20% were on a GLP-1RA. Median UACRs were comparable across arms: 574 (interquartile range, 274–999) with combination therapy, 578 (292–1289) with finerenone, and 583 (301–1114) with empagliflozin. By day 180, the UACR reduction achieved with empagliflozin plus finerenone exceeded that of finerenone alone by 29% (least-squares mean ratio of the difference in change from baseline, 0.71; 95% CI, 0.61 to 0.82; P < 0.001) and that of empagliflozin alone by 32% (least-squares mean ratio, 0.68; 95% CI, 0.59 to 0.79; P < 0.001). The 52% UACR reduction seen with combination therapy at day 180 was consistent with the full additive effect predicted from the individual reductions with finerenone (32%) and empagliflozin (29%) alone (Agarwal et al., 2025a). Taken together, these findings indicate that starting combination therapy early may yield larger and faster reductions in albuminuria than adding agents sequentially.

Additional physiologic benefits further supported the complementary nature of combination therapy. Systolic blood pressure decreased more prominently in the combination-therapy group, with an approximate reduction of 7.4 mmHg within the first 30 days. Reductions in blood pressure were smaller in both monotherapy arms - generally less than half of what combination therapy achieved. Reassuringly, the overall safety profile stayed favorable, with no meaningful differences in serious adverse events across groups. Acute kidney injury events were modestly more frequent with combination therapy, though the absolute rate remained low. Interestingly, the incidence of hyperkalemia was approximately 15%–20% lower in the combination group compared with finerenone alone, potentially reflecting protective tubular and hemodynamic effects of SGLT2 inhibition that may mitigate potassium retention associated with MR blockade (Agarwal et al., 2025a). Similar albuminuria-lowering benefits were observed among participants receiving background GLP1-RA therapy, although no major interaction effect was identified between GLP1-RA use and treatment response (Agarwal et al., 2025b). It is important to highlight that the CONFIDENCE trial primary endpoint was reduction in UACR over 6 months, a surrogate marker over a relatively short observation period. Longer-term data on hard renal outcomes, cardiovascular events, treatment burden, and cost-effectiveness of the finerenone-SGLT2i combination are needed before this strategy can be considered a confirmed standard of care rather than a promising but preliminary approach.

Collectively, these findings suggest a plausible evolving therapeutic paradigm in which DKD management may move beyond isolated single-agent therapy toward integrated multidrug cardiorenal-metabolic protection. However, this remains a hypothesis supported chiefly by surrogate-endpoint data rather than an established treatment strategy: established outcome benefits, reductions in ESKD, dialysis initiation, and cardiovascular death, have been demonstrated for individual agents in large-scale outcome trials, whereas the evidence for combination regimens rests substantially on albuminuria reduction rather than hard endpoints. Until adequately powered long-term outcome trials of combination therapy are available, claims of superior clinical outcomes with multidrug regimens should be regarded as biologically plausible, not yet established.

## 5 From ‘slowing CKD progression’ to ‘achieving CKD remission’

**FIGURE 2:**
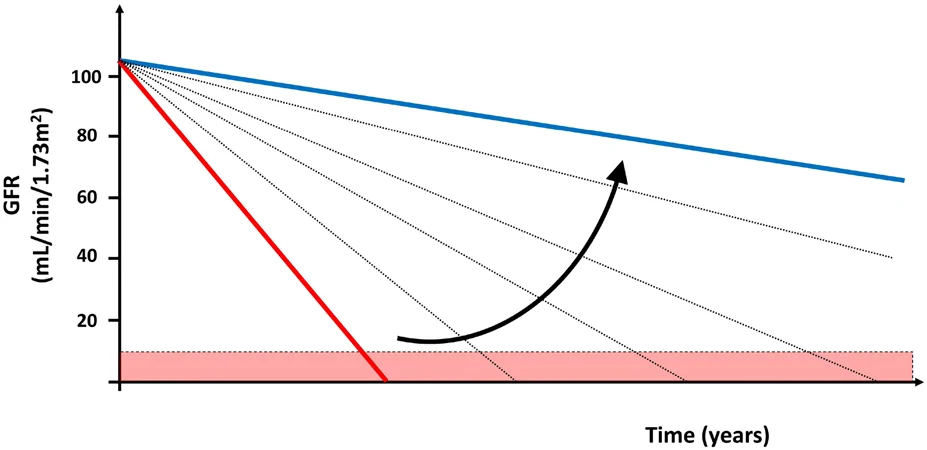
Changing the progression of diabetic kidney disease with new therapeutic advances. This figure illustrates a hypothesis-generating trajectory observed in selected patient subgroups under combination therapy and should not be interpreted as an expected or guaranteed outcome across the general DKD population. The blue line indicates the decline in GFR expected with normal ageing. The red line indicates the decline in GFR that could be expected in DKD. The dashed lines indicate the change in slope of the decline in GFR with the addition of ACE inhibitors/ARBs, SGLT2 inhibitors, GLP-1 RA, and nsMRAs. In theory, the slope could be changed and approximate that of normal ageing defining the concept of ‘CKD remission’. CKD, chronic kidney disease; GFR, glomerular infiltration rate.

The therapeutic advances observed in DKD over the past decade have been so remarkable that nephrology may be approaching a new era in which the trajectory of CKD can be fundamentally altered. Historically, treatment strategies in CKD focused primarily on delaying decline in kidney function and postponing the onset of kidney failure. However, the cumulative benefits achieved through contemporary multidrug cardiorenal-metabolic therapies have begun to shift the therapeutic goal from merely “slowing CKD progression” toward preserving long-term kidney function (Mohsen et al., 2026). This evolving paradigm is supported by substantial reductions in albuminuria, attenuation of eGFR decline, and improvements in CV outcomes observed with renin-angiotensin system blockade, SGLT2 inhibitors, GLP1-RAs, and MRAs. The emergence of multidrug combination therapy strategies further raises the possibility that aggressive early intervention may not only delay CKD progression but potentially stabilize kidney function over prolonged periods. Increasingly, evidence suggests that in selected individuals with DKD, the decline in eGFR may be reduced to rates approaching those expected with physiologic aging alone (Figure 2) (Tangri et al., 2026).

This concept represents a profound shift in the outlook of CKD management. Rather than viewing progressive nephron loss as an unavoidable consequence of DKD, contemporary therapies now raise the possibility of achieving prolonged preservation of kidney function and a state that could reasonably be conceptualized as “CKD remission”. The concept of ‘CKD remission’ broadly reflects stabilization of kidney function, substantial reduction in albuminuria, mitigation of ongoing kidney injury, and prevention of clinically meaningful progression over time. However, this term should be used cautiously and provisionally. At present, no validated, consensus definition exists, and prospective studies with long-term structural and functional outcome data are needed before this terminology can be applied in clinical practice. We see value on this term to describe a hypothesis-generating shift in therapeutic ambition, analogous to remission concepts in oncology and heart failure, rather than a clinically validated endpoint. Importantly, this shift toward CKD remission should not imply cure or reversal of all structural kidney injuries, but rather achievement of disease stability and preservation of functional kidney reserve. Similar to modern approaches in HF and oncology, the focus is increasingly transitioning toward long-term disease modification and maintenance of organ function.

## 6 Limitations and unresolved questions

Several uncertainties are associated with multidrug cardiorenal-metabolic therapy. First, the long-term safety of combining SGLT2 inhibitors, GLP-1 receptor agonists, and non-steroidal MRAs in the same patient has not been established in dedicated long-term safety trials; monitoring burden and cumulative adverse effect risk remain incompletely characterized. Second, non-steroidal MRAs carry a clinically meaningful hyperkalemia risk, particularly in patients with reduced eGFR or those receiving concomitant RAAS inhibition, requiring careful patient selection and monitoring. Third, optimal treatment sequencing, which agent to initiate first, and at what point in the CKD trajectory remains largely empirical rather than evidence-based. Fourth, albuminuria, while a validated and regulatory-accepted surrogate endpoint, does not fully guarantee long-term preservation of hard renal outcomes. Discordance between albuminuria reduction and eGFR trajectory has been observed in some populations, underscoring the need for outcome trials rather than surrogate-endpoint trials alone to confirm the benefit of combination strategies. Finally, cost-effectiveness and accessibility of these therapies, particularly in resource-limited settings, remain substantial barriers to widespread implementation.

## 7 Implementation of combined therapy for diabetic kidney disease in clinical practice

The next major challenge in DKD management is ensuring that these highly effective therapies are successfully incorporated into clinical practice. Growing evidence suggests that a large gap remains between evidence and real-world implementation. Bridging this divide will be essential if the full potential of modern CKM-directed therapy is to be realized at the populational level.

Several studies from diverse healthcare systems consistently demonstrate underutilization of guideline-directed therapies among individuals with DKD. A cross-sectional survey involving approximately 1.2 million veterans in the United States between 2019 and 2020 revealed that GLP1-receptor agonists and SGLT2 inhibitors were prescribed in only 12% and 10%, respectively, of veterans with concomitant diabetes and CKD (Lamprea-Montealegre et al., 2022). Similarly, electronic medical record data from two large healthcare centers in the United States involving more than 39,000 adults with diabetes and CKD between 2019 and 2020 demonstrated prescription rates of only 40.4% for ACE inhibitors/ARBs, 5% for SGLT2 inhibitors, and 6.3% for GLP1-RA therapy during follow-up (Nicholas et al., 2023).

Additional evidence from large healthcare networks further highlights this implementation gap. A retrospective cohort study involving 20 healthcare systems participating in the United States National Patient-Centered Clinical Research Network demonstrated that among adults with type 2 diabetes, CKD, and albuminuria between 2015 and 2020, 78% received ACE inhibitors or ARBs, only 4.6% received SGLT2 inhibitors, and 21% received neither therapy (Edmonston et al., 2024). Similar findings have been observed internationally. A cross-sectional study of more than 500,000 adults with CKD in England demonstrated that although over one-quarter of the CKD population met guideline-directed indications for SGLT2 inhibitor therapy, only 17% of eligible individuals were prescribed these medications, with utilization occurring predominantly among those with coexisting type 2 diabetes (Forbes et al., 2024).

Underutilization of contemporary CKM therapies has also been demonstrated in Australian and Danish cohorts. In a retrospective cohort study involving more than 114,000 adults with type 2 diabetes in Australia, SGLT2 inhibitors were prescribed in only 14.4% of patients with CKD, while GLP1-RA therapy was prescribed in only 10.1% (Wallace et al., 2025).

Similarly, the ReDiCare project evaluating hospitalized Australian patients with type 2 diabetes and CKD found that only 21.3% received SGLT2 inhibitors, 7.4% received GLP1-RA therapy, and merely 0.6% received all four major cardiorenal protective drug classes despite broad evidence supporting multidrug therapy (Lan et al., 2025). National registry data from Denmark demonstrated increasing utilization of SGLT2 inhibitors and GLP1-RA over time; however, use remained significantly lower among individuals with CKD compared with those without CKD despite these patients representing the highest-risk population (Rørth et al., 2025).

Collectively, these findings demonstrate that implementation of multidrug CKM therapy remains profoundly inadequate worldwide. Multiple barriers likely contribute to this gap, including therapeutic inertia, concerns regarding polypharmacy and adverse effects, medication cost, limited familiarity with newer therapies, fragmented multidisciplinary care, delayed nephrology referral, and uncertainty regarding sequencing or combination of therapies. Importantly, the patients who stand to benefit the most from these interventions, those with advanced CKD, albuminuria, CV disease, and high cardiorenal risk, often remain undertreated.

The current implementation gap in nephrology parallels challenges previously encountered in HF management. Over the past several decades, cardiology societies have developed structured quality improvement initiatives aimed at increasing implementation of guideline-directed medical therapy for HF. Programs such as Get With The Guidelines have substantially improved adherence to evidence-based therapies and contributed to improved patient outcomes in HF populations (Tang et al., 2025). In HF management, delays in initiation of evidence-based therapies are increasingly recognized as exposing patients to preventable morbidity and mortality. A similar sense of urgency is likely needed in DKD management.

The implementation barriers described have been characterized predominantly in high-income healthcare systems with established specialist nephrology and cardiology infrastructure. In many low- and middle-income countries, additional and often more fundamental barriers exist, including limited medication affordability, restricted access to SGLT2 inhibitors and GLP-1 receptor agonists due to cost, scarcity of nephrology and endocrinology specialists, and inconsistent CKD screening. Addressing the global DKD burden will require implementation strategies tailored to local resource availability, potentially prioritizing lower-cost agents (e.g., generic SGLT2 inhibitors where available) and task-shifting screening and initial management to primary care and non-physician providers.

## 8 Final thoughts

Recent progress in how DKD is understood, prognosticated, and treated has fundamentally reshaped the field of nephrology. It is becoming clear that slowing CKD progression and reducing CV risk cannot be viewed as separate therapeutic objectives. The CKM construct helps unify nephrology and cardiology by recognizing the simultaneous kidney and CV risks among those with DKD and emphasizing the need for integrated multidisciplinary care. This reframing of DKD within the CKM framework represents a major therapeutic paradigm shift. Historically, management strategies focused primarily on glycemic control and delaying progression to kidney failure. However, contemporary therapies now simultaneously improve kidney and CV outcomes. ACE inhibitors/ARBs, SGLT2 inhibitors, GLP1-RAs, and MRAs collectively target complementary pathways involved in DKD progression. These therapies not only reduce albuminuria and slow decline in eGFR, but also significantly reduce hospitalization for HF, major adverse CV events, and mortality.

In parallel with the “Fantastic Four” concept in HF with reduced ejection fraction (Bauersachs, 2021), these contemporary therapies have increasingly been referred to as the “Fantastic Four in Nephrology” (Mima, 2022). Although this terminology reflects the growing enthusiasm surrounding multidrug cardiorenal protection, it also highlights the evolving recognition that comprehensive CKM-directed therapy may provide additive and potentially synergistic benefits. Importantly, the CONFIDENCE trial demonstrated substantial additive reductions in albuminuria with combined finerenone and empagliflozin therapy, supporting the rationale for multidrug approaches. Nevertheless, whether all modern therapies exert fully additive effects remains incompletely understood. For example, *post hoc* analyses from CONFIDENCE did not clearly demonstrate additional benefits attributable to baseline GLP1-RA therapy, suggesting that the optimal sequencing, timing, and integration of multidrug therapy still requires further investigation. Although the “Fantastic Four” concept provides an appealing and clinically practical framework, the field is still evolving, and caution should be exercised before assuming additive benefit across all therapeutic combinations.

Importantly, the rapid pace of therapeutic innovation suggests that the current treatment paradigm may continue to evolve substantially over the coming years. Emerging therapies including aldosterone synthase inhibitors and endothelin A receptor antagonists have shown promising kidney and CV effects and may further expand the therapeutic armamentarium available for DKD management (Heerspink et al., 2023; Tuttle et al., 2024). Despite these advances, major challenges remain regarding implementation of evidence-based therapies into clinical practice. The persistent gap between evidence and real-world highlights the urgent need for multidisciplinary CKM-focused care models aimed at improving adoption of guideline-directed therapy. Ultimately, the success of the CKM paradigm will depend not only on the development of novel therapeutics, but also on the ability to effectively deliver integrated cardiorenal-metabolic care to the growing population of patients with DKD.

**FIGURE 3:**
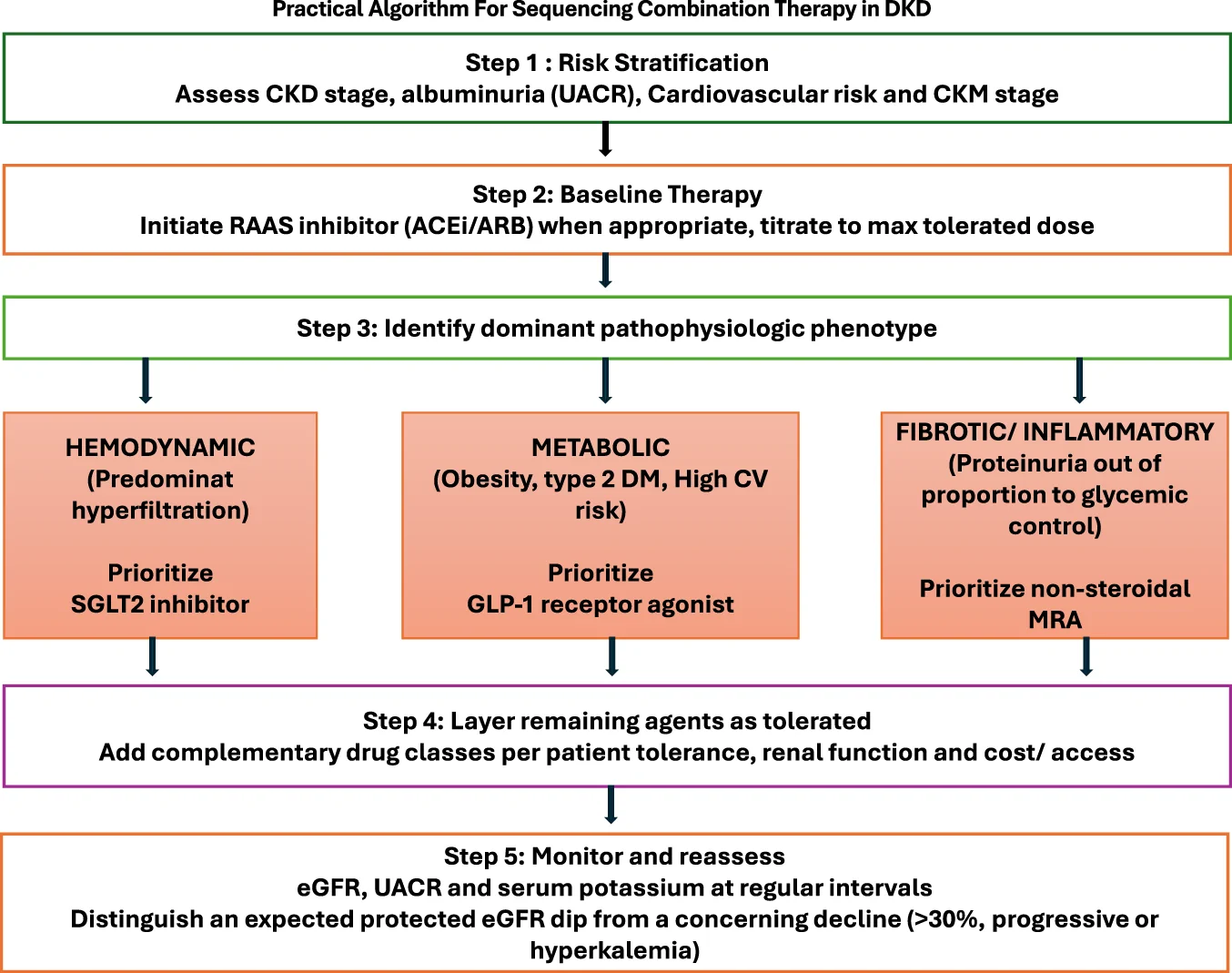
Practical algorithm for sequencing combination therapy in DKD. ACEi, angiotensin-converting-enzyme inhibitor; ARB, angiotensin receptor blocker; CKD, chronic kidney disease; CKM, cardio-kidney-metabolic; CV, cardiovascular; DKD, diabetic kidney disease; DM, diabetes mellitus; eGFR, estimated glomerular filtration rate; MRA, mineralocorticoid receptor antagonist; RAAS, renin-angiotensin-aldosterone system; UACR, urine albumin-to-creatinine ratio.

Current combination-therapy strategies are based mostly on an uniform sequence of add-on therapies irrespective of individual patient phenotype. We propose that the dominant pathophysiologic driver in each patient, hemodynamic (intraglomerular hypertension), metabolic (hyperglycemia-driven hyperfiltration), or fibrotic/inflammatory (mineralocorticoid-driven mesangial injury), should inform which agent should be prioritized first, rather than defaulting to a fixed sequence. For example, a patient with proteinuria disproportionate to glycemic control may derive earlier benefit from MRA-driven anti-fibrotic action, while a patient with hyperfiltration and elevated filtration fraction may benefit from early SGLT2 inhibitor-driven afferent arteriole constriction (Figure 3). This phenotype-driven framework does not yet have prospective validation, but it reframes combination therapy from a sequential checklist to a mechanistically guided, individualized strategy. Prospective studies stratifying patients by dominant pathophysiologic phenotype will be needed to validate whether this improves outcomes.
